# Breaking glycolysis: allosteric hotspots for multi-target drug repurposing

**DOI:** 10.1007/s10822-026-00830-5

**Published:** 2026-05-16

**Authors:** Latife Sude Vural, Elcin Kahraman, Simay Mintemur, Sinem Urhan, E. Demet Akten

**Affiliations:** 1 Graduate Program of Computational Sciences and Engineering, Graduate School of Science and Engineering, Kadir Has University, Istanbul, Turkey; 2 Department of Molecular Biology and Genetics, Faculty of Engineering and Natural Sciences, Kadir Has University, Istanbul, Turkey

**Keywords:** Glycolysis, Allosteric regulation, Staphylococcus aureus, Drug repurposing, species-specificity, polypharmacology, virtual screening, elastic network model

## Abstract

Glycolysis is essential for cellular energy production, making its enzymes attractive antimicrobial targets. Traditional strategies targeting conserved catalytic sites risk host toxicity due to limited species specificity. Allosteric sites—spatially distinct, evolutionarily divergent regulatory regions—offer selective inhibition but are challenging to detect experimentally. Here, we systematically mapped allosteric sites across all ten glycolytic enzymes of *Staphylococcus aureus* and *Plasmodium vivax* using a multi-scale computational framework combining elastic network models, residue interaction networks, and complementary machine-learning algorithms. High-confidence allosteric sites were identified in fructose-1,6-bisphosphate aldolase, triosephosphate isomerase, and phosphoglycerate mutase, revealing diverse regulatory architectures, from interfacial network hubs to hinge-mediated dynamic control. Experimentally validated sites in pyruvate kinase and phosphofructokinase further reinforced the predictive framework. Comparative analysis with human homologs confirmed pronounced species-specific divergence, supporting selective targeting. Virtual screening 1,615 FDA-approved compounds across all ten enzymes identified multi-target ligands exhibiting amphiphilic, interface-stabilizing architectures capable of coordinated glycolytic modulation. Binding analyses revealed a balanced contribution of polar and hydrophobic interactions, consistent with robust allosteric modulation. This pathway-wide, network-informed approach demonstrates the feasibility of selective disruption of bacterial glycolysis and provides a blueprint for rational polypharmacology and next-generation antimicrobial design.

**Supplementary Information:**

The online version contains supplementary material available at 10.1007/s10822-026-00830-5.

## Introduction

Glycolysis is a central metabolic pathway responsible for ATP production and carbon flux in all living organisms, comprising ten conserved enzymatic reactions that initiate glucose catabolism. Because glycolytic enzymes are essential for cellular viability, they have long been considered promising targets for antimicrobial drug development [1, 2]. However, the strong evolutionary conservation of their catalytic sites severely limits target selectivity and increases the risk of host toxicity when inhibitors interact with homologous human enzymes [3]. This challenge highlights the need for alternative strategies capable of selectively targeting pathogen-specific regulatory features of essential metabolic enzymes.

In contrast to catalytic sites, allosteric sites represent ligand-binding regions spatially distinct from the catalytic center that modulate enzyme activity through long-range conformational coupling. As a result, these regulatory regions tend to exhibit greater sequence and structural divergence across species, making them attractive targets for selective drug design. Importantly, many allosteric sites are transient, cryptic, or conformationally regulated, and therefore difficult to detect using classical experimental approaches alone [4, 5]. Recent advances in structural biology and computational methods, including molecular dynamics simulations, residue network analysis, and structure-based pocket detection, now enable systematic identification and characterization of such noncanonical regulatory sites. Leveraging these approaches to map and compare allosteric landscapes of glycolytic enzymes across species offers a promising strategy to uncover selective, species-specific druggable sites and further strengthens the foundation for rational antimicrobial design [4, 6, 7].

Allosteric regulation is inherently dynamic and often mediated by long-range communication between distal residues rather than by static binding pockets. Elastic Network Models (ENMs) and residue interaction network analyses provide efficient and physically grounded frameworks for identifying collective motions and information-flow pathways that underlie allosteric regulation in proteins [8, 9]. By representing proteins as networks of interacting residues connected through C^α^ atoms, these coarse-grained models capture large-scale conformational fluctuations that govern functional regulation. Such approaches allow the identification of key communication hubs, dynamically coupled regions, and evolutionarily divergent potential regulatory nodes that may not be evident from static structural analyses alone [8–10].

In our previous study by Ayyildiz et al. [11], computational solvent mapping was first employed to identify potential binding sites, followed by the ENM-based residue scanning method ESSA [36] to distinguish putative allosteric regions in phosphofructokinase (PFK), glyceraldehyde-3-phosphate dehydrogenase (GAPDH), and pyruvate kinase (PK). These predictions were further supported by complementary evaluation metrics from DoGSiteScorer [12] and AlloSigMA [13], which assess pocket druggability and allosteric signaling, respectively. Notably, several of the identified regions overlapped with experimentally characterized regulatory sites, providing support for the validity of the computational approach.

In a subsequent study by Alnigenis et al. [14], the same framework was extended to four additional glycolytic enzymes—hexokinase, phosphoglucose isomerase (PGI), phosphoglycerate kinase (PGK), and enolase—where computational solvent mapping was combined with CavityPlus to identify druggable pockets. The detected sites were then systematically evaluated using multiple complementary methods, including ENM-based approaches (ESSA, CorrSite), energetics-based AlloSigMA, and the machine-learning-based predictor PASSer, thereby improving robustness, reducing method-specific bias, and enabling cross-validation of predicted allosteric regions.

The present study further extends this integrated computational framework with the addition of an interaction network-based tool Ohm [15] to the remaining three glycolytic enzymes—fructose-1,6-bisphosphate aldolase (FBA), triosephosphate isomerase (TIM), and phosphoglycerate mutase (PGM)—thereby completing a comprehensive, pathway-wide mapping and prioritization of potential allosteric control points in glycolysis [16–19].

The bacterial pathogen *Staphylococcus aureus* was selected as the target organism due to its high clinical relevance and its well-documented capacity to develop antibiotic resistance [20]. Species-specific drug design aims to selectively inhibit pathogen enzymes while minimizing off-target effects on human homologs, an objective more readily achieved by targeting less conserved allosteric sites rather than highly conserved catalytic residues [16]. Although experimental studies have established the existence of allosteric regulation in several glycolytic enzymes, systematic computational analyses comparing species-specific allosteric landscapes remain limited [7].

A notable observation across these enzymes is the limited availability of direct biophysical validation of ligand binding to proposed allosteric regions, particularly those located at interfacial or hinge sites. For example, in TIM, several studies have identified small molecules targeting the dimer interface and demonstrated their functional effects through enzyme inhibition assays and molecular dynamics analyses; however, direct quantification of binding affinity using techniques such as surface plasmon resonance (SPR) or isothermal titration calorimetry (ITC) has not been widely reported [21–23]. Similarly, in GAPDH, although biophysical methods such as ITC and thermal shift assays have been applied to characterize ligand binding, these studies predominantly focus on the catalytic or cofactor-binding sites rather than distal allosteric or interfacial pockets [24, 25]. For enzymes such as aldolase, PGM, and PGK, the existence of allosteric regulation is largely supported by structural and dynamical analyses, with minimal direct evidence of ligand binding at proposed regulatory regions [17–19]. Enolase, likewise, is primarily regulated through metal ion coordination and protein–protein interactions, with no well-established small-molecule allosteric binding sites validated through biophysical measurements.

To address this gap, we employed a comprehensive computational workflow to identify and characterize putative allosteric binding pockets in S. *aureus* FBA, TIM, and PGM [26]. Following identification of these regulatory regions, a structure-based virtual screening was performed to evaluate FDA-approved compounds for their potential to interact with the predicted allosteric pockets. Rather than focusing on inhibition of a single enzymatic target, candidate molecules were prioritized based on their collective binding affinities across all ten glycolytic enzymes. By integrating docking results across the entire pathway, we identified several FDA-approved compounds that consistently engage multiple predicted allosteric sites in the glycolytic network. This multi-target strategy is designed to perturb the glycolytic pathway more effectively than conventional single-enzyme inhibition [27].

The goal of this study was therefore to determine whether less-conserved allosteric sites in *S. aureus* glycolytic enzymes can serve as selective targets for species-specific inhibitor design while minimizing interactions with human homologs. By integrating protein dynamics, allosteric site prediction, and virtual screening, this work establishes a comprehensive computational framework for the rational discovery of novel antibacterial therapeutics targeting central carbon metabolism. Also, by framing glycolysis as an interconnected regulatory network rather than a collection of isolated enzymes, this study provides a systems-level framework for pathway-wide antimicrobial targeting.

## Materials and methods

**Fig. 1:**
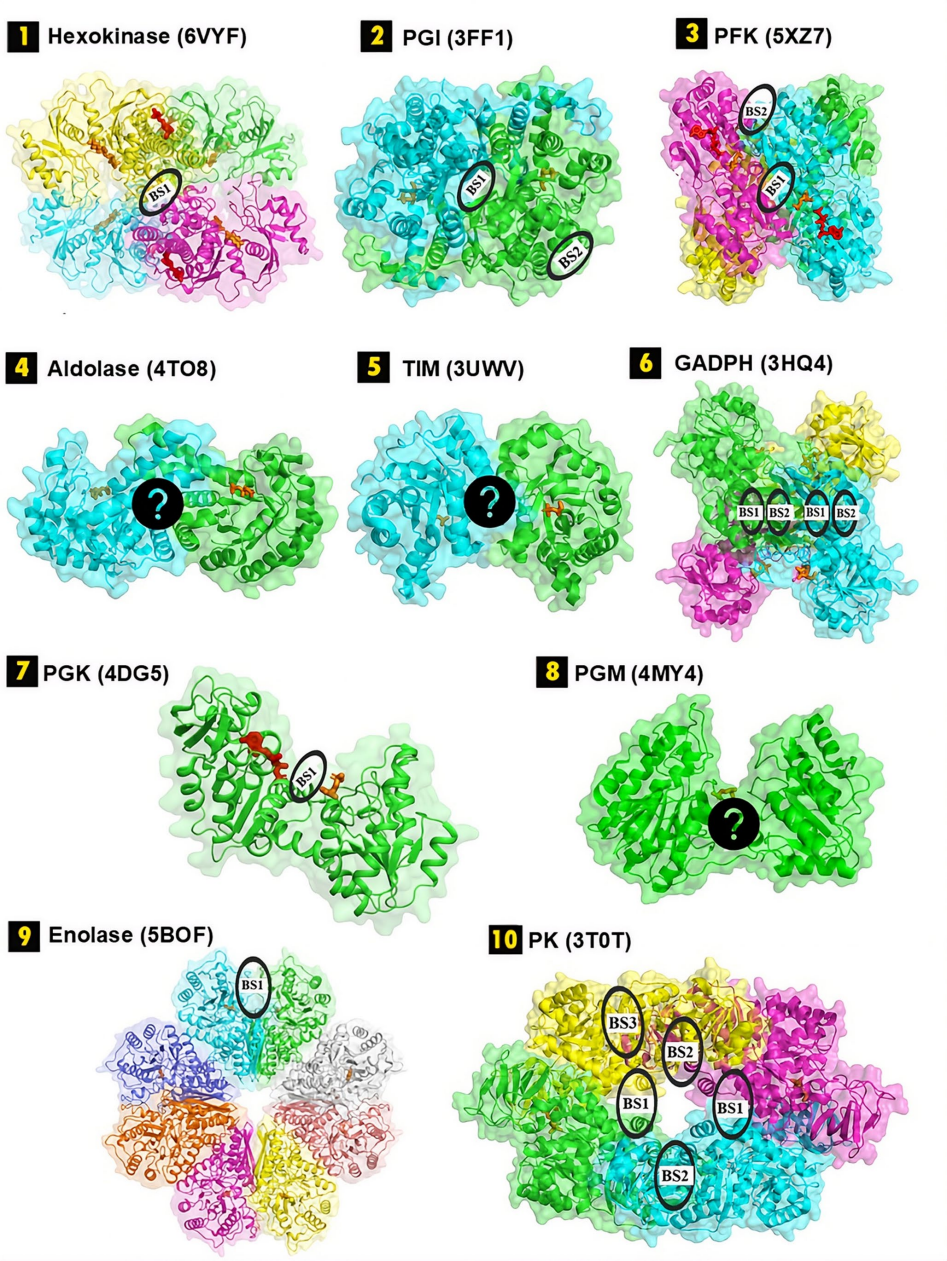
3D structure of ten glycolytic enzymes. Seven of them are illustrated with their potential allosteric binding sites (BS) identified in previous works [11, 14], while the remaining three enzymes have unresolved allosteric sites

This study investigates potential allosteric sites in three glycolytic enzymes—fructose-1,6-bisphosphate aldolase (FBA), triosephosphate isomerase (TIM), and phosphoglycerate mutase (PGM)—using an integrated computational framework. As illustrated in Fig. 1 and summarized in Table 1, similar analyses conducted for the remaining seven glycolytic enzymes in previous studies [24, 25, 28, 29] identified several allosteric pockets. The same analytical pipeline was applied to the three remaining enzymes through four stages: (i) identification of druggable binding sites, (ii) evaluation of allosteric propensity, (iii) assessment of species specificity, and (iv) virtual screening of FDA-approved compounds.

**Table 1:** List of glycolytic enzymes under study

Enzyme	PDB ID	Species	Biological assembly	Total # of residues	Substrate	Cofactor
1. Hexokinase	6VYF	*P. Vivax*	Homotetramer	1728	Glucose	ATP
2. PGI	3FF1	*S. aureus*	Homodimer	888	G6P	–
3. PFK	5XZ7	*S. aureus*	Homotetramer	1304	F6P	ATP
4. Aldolase	4TO8	*S. aureus*	Homodimer	557	F1,6BP	–
5. TIM	3UWV	*S. aureus*	Homodimer	509	DHAP	–
6. GAPDH	3HQ4	*S. aureus*	Homotetramer	1336	G3P	NAD^+^
7. PGK	4DG5	*S. aureus*	Monomer	405	1,3-BPG	ADP
8. PGM	4MY4	*S. aureus*	Monomer	503	3-PG	–
9. Enolase	5BOF	*S. aureus*	Homooctamer	3536	2-PG	–
10. PK	3T0T	*S. aureus*	Homotetramer	2332	PEP	ADP

###  Step 1. Identification of druggable binding sites

The receptor surface was systematically analyzed for potential ligand-binding pockets using FTMap, a computational solvent-mapping tool that conceptually mimics X-ray crystallographic fragment screening [30]. Sixteen small organic probe molecules were independently docked onto the receptor via energy minimization. Regions where multiple probe molecules of different chemical compounds converged were identified as “*hot spots*”, indicating structurally and energetically favorable binding sites. To improve conformational sampling, an ensemble of receptor conformers generated by ClustENMD [31], based on the elastic network model, was analyzed using FTMove [32], an extension of FTMap designed for handling multiple conformers. In addition, CavityPlus [33] was employed to further identify potential binding pockets based on geometric and physicochemical features, including cavity volume, hydrophobic volume, surface area, lip size, and hydrogen bonding potential.

###  Step 2. Evaluation of allosteric properties of binding sites

Binding sites identified in the initial screening were subsequently evaluated for allosteric properties using an integrated, multi-scale analysis framework. Structural, dynamic, network-based, and energetic properties of candidate sites were assessed using five complementary methods: CorrSite [34], OHM [35], ESSA [36], PASSer [37], and AlloSigMA [13].

OHM (Optimal Hierarchical Method) characterizes allosteric communication by modeling the protein as a weighted residue interaction network and computing residue-level allosteric coupling intensities (0–1) based on graph-matching and signal propagation [15]. Dynamic sensitivity was evaluated using ESSA (Essential Site Scanning Analysis), which applies localized perturbations to elastic network models and quantifies the resulting redistribution of low-frequency collective modes; perturbation-induced eigenvalue shifts were Z-score normalized to identify residues exerting strong control over global dynamics [36].

Structural propensity for allostery was assessed using PASSer (Protein Allosteric Sites Server), a machine learning–based method that integrates pocket geometry and physicochemical descriptors to rank candidate allosteric sites [37]. Finally, AlloSigMA was used to quantify the energetic consequences of perturbations by computing residue-level changes in conformational free energy within an elastic network–based statistical mechanical framework [13]. Together, these methods provide a robust and complementary characterization of allosteric sites and communication pathways.

###  Step 3. Assessment of species specificity

Following evaluation of their allosteric predisposition, bacterial enzymes were compared with their human homologs to assess sequence and structural conservation within the putative allosteric binding pockets. Global pairwise sequence alignments were performed using the Needleman–Wunsch algorithm [38] to compute sequence identity and similarity percentages between species. Structural alignments were conducted in PyMOL [39], and structural similarity of the predicted allosteric sites was quantified by calculating root-mean-square deviation (RMSD) values for Cα atoms within the corresponding pocket residues.

###  Step 4. Repurposing FDA compounds to putative allosteric sites

Binding sites exhibiting strong likelihood of allosteric regulation and species specificity were subsequently subjected to molecular docking analysis. Docking calculations were performed using the GOLD software tool with the ChemPLP scoring function, which has demonstrated strong performance in predicting protein-ligand binding modes and affinities [40, 41]. Compound prioritization was then based on an integrated analysis combining these results with previously reported docking data for the remaining seven glycolytic enzymes. From a library of 1,615 FDA-approved compounds extracted from the ZINC15 database [42], molecules exhibiting binding affinity across all ten enzymes were identified as lead candidates for subsequent experimental validation. Molecules exhibiting favorable binding across all ten enzymes were identified as candidate multi-target inhibitors for further experimental validation. FDA-approved compounds were retrieved from the ZINC database by selecting entries annotated as approved drugs within the ZINC15 “FDA-approved” subset. The compounds were downloaded in [specify format, e.g., MOL2/SDF] format for subsequent preparation and docking.

The screening library comprised 1,615 FDA-approved compounds spanning well-established drug-like chemical space. The molecular weight distribution predominantly fell within the range of approximately 200–500 Da, with a central tendency around 350–450 Da. Lipophilicity values (logP) were generally moderate, clustering between 2 and 4, consistent with a balance between aqueous solubility and membrane permeability. The topological polar surface area (TPSA) values were generally below 120 Å², indicating favorable oral bioavailability for most compounds. Hydrogen-bonding capacity was within conventional limits, with most molecules possessing no more than five hydrogen-bond donors and ten hydrogen-bond acceptors. Molecular flexibility was moderate, as reflected by a typical range of fewer than ten rotatable bonds. However, a subset of highly flexible compounds was also included to preserve chemical diversity. Overall, the library exhibits physicochemical characteristics consistent with Lipinski’s Rule of Five and related drug-likeness criteria, supporting its suitability for multitarget virtual screening and drug repurposing applications.

## Results and discussion

### Part I. Identification of species-specific allosteric sites in three glycolytic enzymes

**Fig. 2:**
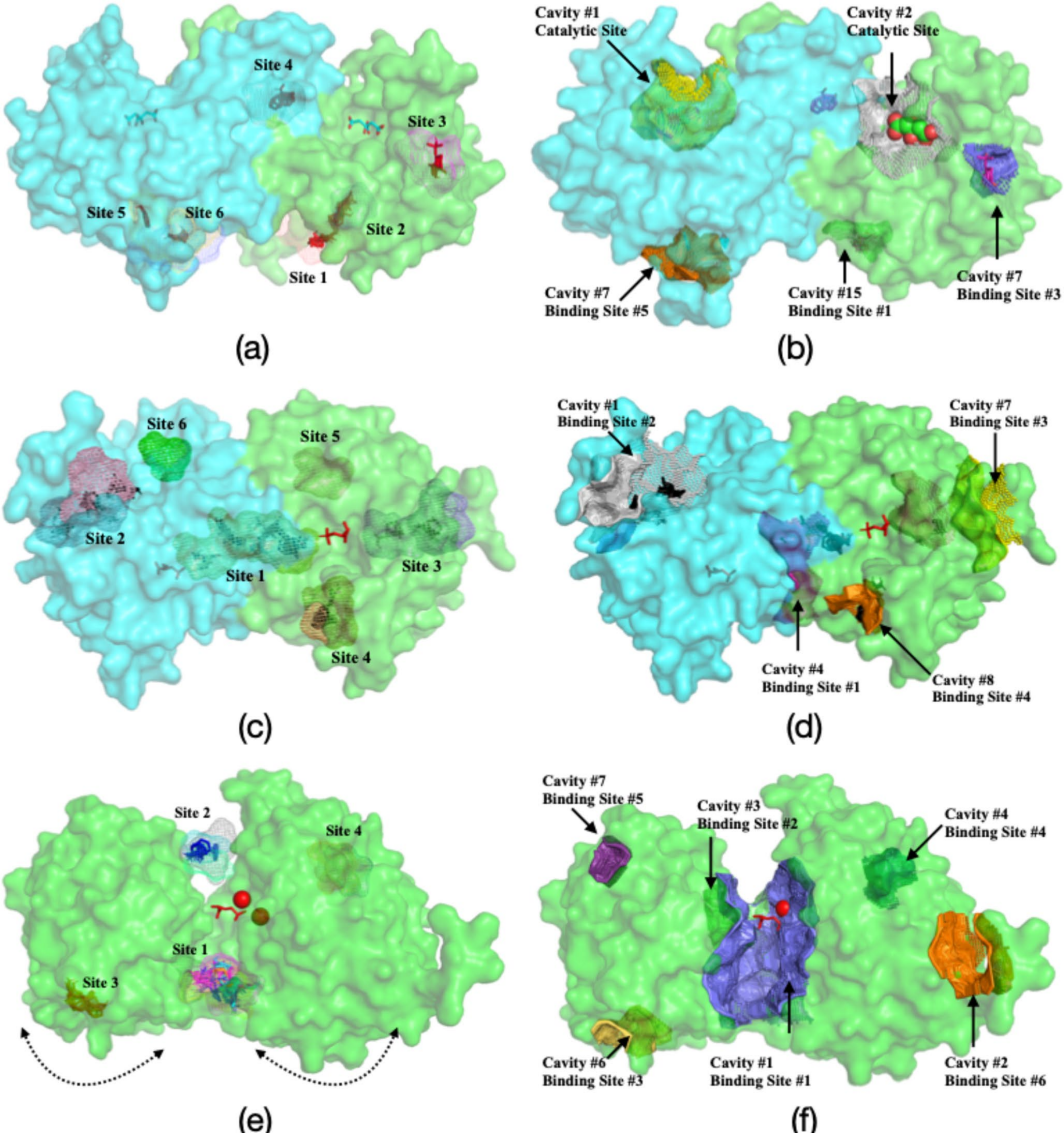
FTMap/FTMove/CavityPlus results of fructose 1,6-bisphosphate aldolase, triosephosphate isomerase, and phosphoglycerate mutase

As illustrated in Fig. 2, FTMap and FTMove identified multiple putative binding sites on the surface of each enzyme. In both dimeric aldolase and TIM (Fig. 2a and c), one of the putative sites (Site #4 in aldolase, Site #1 in TIM) was observed at the dimer interface, a region commonly associated with allosteric regulation, supporting its potential functional relevance. For the monomeric enzyme PGM, a prominent hot spot (Site #1) was detected at the hinge region, which exhibits characteristic opening–closing dynamics, as indicated by arrows (See Fig. 2e). The use of an ensemble of conformations, rather than a single static structure, increased the number of detected binding sites: additional sites (#5 and #6) were identified for TIM, and Site #4 emerged for PGM, as depicted in Fig. 2d and f, respectively. In contrast, no additional binding sites were detected for aldolase.

**Fig. 3:**
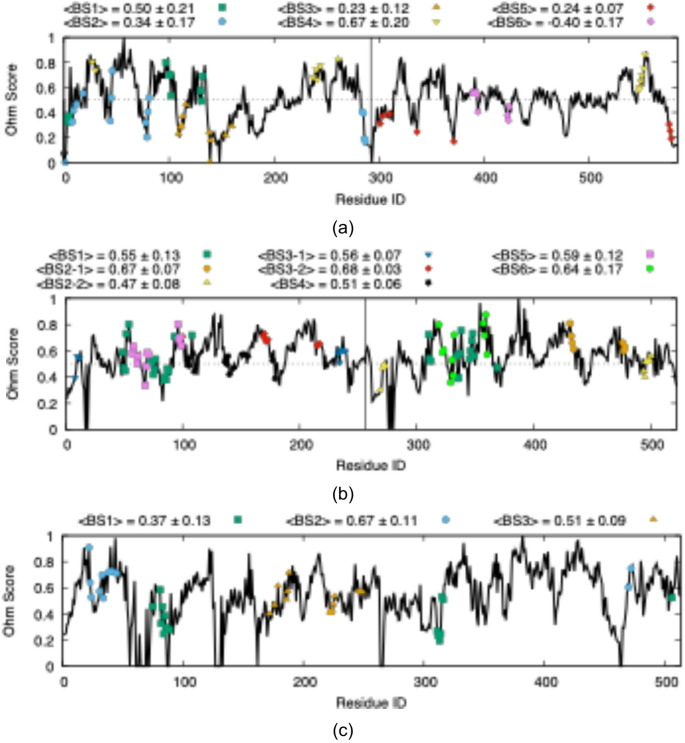
Residue-based Ohm score values for **a** fructose 1,6-bisphosphate aldolase, **b** triosephosphate isomerase, and **c** phosphoglycerate mutase

The allosteric propensity of each binding site was quantified using the allosteric coupling intensity (ACI), a network-based metric that captures the frequency with which residues respond to a perturbation. As shown in Fig. 3, an ACI threshold of 0.5 was used to delineate putative allosteric hot spots with the capacity to efficiently transmit signals to distal functional regions of the enzyme. For each site, a mean ACI value was computed over the constituent residues (See Supplementary Table 1). In aldolase, binding sites #1 and #4, either located at or near the inter-subunit interface, exceeded this threshold, providing a structural rationale for their strong allosteric predisposition. Triosephosphate isomerase, by comparison, exhibited uniformly high ACI values across nearly all identified sites, consistent with its extensive dynamic and allosteric communication network reported for this enzyme, particularly involving coordinated motions of catalytic loops and inter-subunit regions [21–23]. In contrast, PGM displayed a more localized allosteric architecture, with only binding sites #2 and #3 exceeding the defined threshold. Notably, binding site #2 was detected exclusively in the closed, substrate-bound conformation of the enzyme, positioning it at the enzyme-substrate interface. This conformation-specific emergence suggests that perturbations at this site may selectively interfere with substrate engagement, thereby providing a mechanistic basis for allosteric inhibition by modulating catalytic dynamics rather than global structural disruption. This regulatory mechanism differs from that observed in aldolase, where high-ACI binding sites are primarily located at inter-subunit interfaces and remain accessible across conformational states. In contrast, PGM relies on a more constrained, dynamics-driven mechanism in which allosteric control is exerted through transient, catalytically coupled sites. These distinctions highlight fundamentally different allosteric strategies and underscore how enzyme architecture dictates both the mechanism and selectivity of allosteric inhibition.

**Fig. 4:**
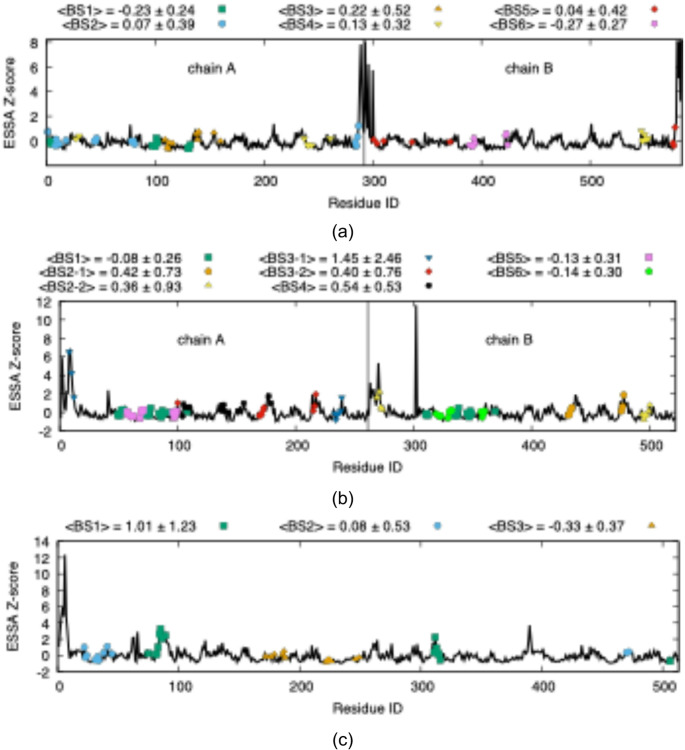
Residue-based ESSA Z-score values for **a** fructose 1,6-bisphosphate aldolase, **b** triosephosphate isomerase, and **c** phosphoglycerate mutase

Another critical assessment of allosteric likelihood was obtained by ESSA, which quantifies the frequency of global motions derived from elastic network models. High ESSA Z-scores identify regions whose perturbation is predicted to exert a change in the collective protein dynamics. Because ESSA scores are normalized, they enable enzyme-specific ranking of binding sites and facilitate meaningful comparison across different enzymes. As shown in Fig. 4a, aldolase binding sites #1, #4, and #6 exhibited the highest ESSA Z-scores, reinforcing the recurrent identification of sites #1 and #4 as dominant allosteric hot spots and underscoring their strong coupling to global motions. In TIM, elevated Z-scores were observed across nearly all binding sites, consistent with its densely connected dynamic network and in agreement with the uniformly high allosteric coupling intensities identified by Ohm (See Fig. 4b). In PGM, binding site #1, which corresponds to the hinge region, exhibited one of the highest Z-scores (Z > 1) as illustrated in Fig. 4c, highlighting its pronounced influence on global dynamics despite its more modest network-based coupling previously identified by Ohm. This discrepancy suggests that hinge-mediated control in PGM operates primarily by modulating large-scale conformational transitions rather than through extensive residue-to-residue communication, revealing a dynamics-driven allosteric mechanism that purely network-centric approaches may underestimate.

**Fig. 5:**
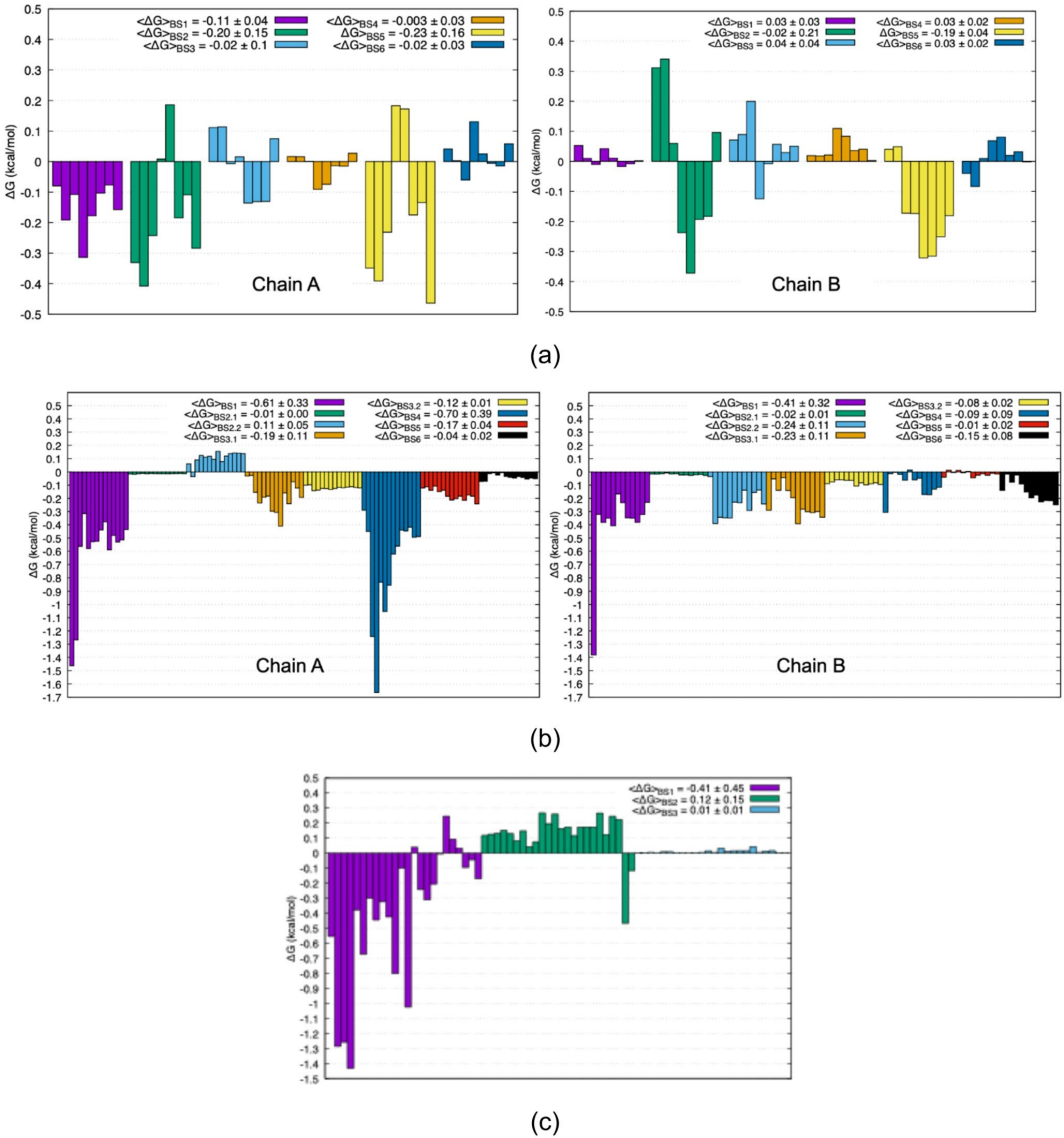
Residue-based AlloSigMA ΔG values for **a** fructose 1,6-bisphosphate aldolase, **b** triosephosphate isomerase, and **c** phosphoglycerate mutase

AlloSigMA provided an additional, energetics-based evaluation of allosteric property by quantifying residue-level conformational free-energy changes within an elastic network–based statistical mechanical framework. In this analysis, negative free energy changes indicate reduced flexibility at the catalytic site upon perturbation of a distal binding site, a response commonly associated with diminished catalytic activity. The analysis requires the knowledge of all substrate and cofactor binding site residues, which were listed for all ten glycolytic enzymes in Supplementary Table 2. As shown in Fig. 5a, aldolase exhibited chain-specific allosteric responses, reflecting asymmetric coupling within its oligomeric assembly. Binding sites #2 and #5 produced the strongest overall effects on both catalytic sites; however, in binding site #2, opposing residue-level contributions largely canceled each other, resulting in a near-zero net response. Binding site #1 selectively perturbed the catalytic site on a single chain. In contrast, binding site #4 had minimal impact on catalytic-site flexibility, despite its strong network-based coupling, suggesting a decoupling between communication efficiency and energetic control at this site.

In TIM, comparatively large free energy changes were observed across nearly all binding sites, with particularly pronounced effects at binding sites #1 and #4, consistent with a globally coupled dynamic architecture in which distal perturbations readily influence catalytic flexibility.

In contrast, PGM displayed a more focused energetic response, with the strongest effect arising from binding site #1, corresponding to the hinge region that governs enzyme opening and closure during activation. The pronounced energetic sensitivity of this site reinforces the notion that allosteric regulation in PGM is predominantly mediated through modulation of large-scale conformational transitions rather than through distributed network interactions.

Final evaluation of binding-site allosteric capacity was performed using PASSer, a machine learning–based approach that integrates pocket geometry with physicochemical descriptors. For aldolase, PASSer identified 12 pockets with probability scores exceeding 10% (Table 2), four of which overlapped with previously defined binding sites, indicating partial convergence between data-driven and physics-based predictions. Notably, binding sites #1 and #3 were not captured among the top-ranked PASSer pockets, suggesting that their allosteric influence may arise primarily from network connectivity and dynamic coupling rather than from intrinsic pocket features. In TIM, five of the eight predicted binding sites ranked within the top ten PASSer pockets, all exceeding the 10% probability threshold. Binding site #1, located at the inter-subunit interface, displayed the highest probability score (46.85%) observed for TIM, reinforcing its strong allosteric predisposition and highlighting the functional importance of quaternary-structure–mediated regulation in this enzyme. In PGM, seven pockets surpassed the 10% threshold, among which binding site #1 corresponding to the hinge region exhibited the highest probability of allosteric behavior (39.78%), representing the strongest PASSer signal observed for this enzyme. The consistent prioritization of this hinge site across machine learning, energetic, and dynamics-based analyses underscores its central role in controlling PGM activation through modulation of conformational transitions.

All evaluation results were combined in Table 2, which also reports the number of probe molecules identified at each site by FTMap. All selected sites were independently evaluated by FTMove across multiple protein conformations, supporting their robustness to conformational variability. CavityPlus either detected the same regions and classified their druggability as strong, medium, or weak, or failed to identify them, while CorrSite similarly assessed their allosteric propensity or omitted them entirely. Quantitative assessments from Ohm, ESSA, and AlloSigMA provided complementary network-, dynamics-, and energetics-based scores for each site, whereas PASSer assigned probability scores only to pockets it explicitly detected.

Integrating these statistically independent metrics, binding sites were prioritized based on convergence across methods, which reinforces the likelihood of allosteric coupling. For aldolase, binding site #1, located proximal to the subunit interface, and binding site #4, positioned directly at the inter-subunit interface and comprising residues from both chains, emerged as the most compelling targets and were therefore selected for screening against FDA-approved compounds for allosteric inhibitors. For TIM, binding site #1, an established interfacial allosteric site [23], was prioritized alongside binding site #2, situated at a distal region of a single chain. Despite its peripheral location, this second site was consistently ranked highly by Ohm, PASSer, ESSA, and AlloSigMA, thus indicating substantial allosteric property. Finally, for PGM, binding site #1, corresponding to the hinge region, was selected as the primary allosteric target because it combines relatively strong druggability with pronounced allosteric and dynamic effects, consistent with its central role in enzyme activation.

**Table 2:** All score values for all binding sites (BS). Binding sites selected for docking experiments are shown in bold.

Enzyme	BS	FTMap	FTMove	Cavity+	CorrSite	OHM	PASSer	ESSA	AlloSIGMa
Aldolase	1*	**14**	**+**	**Weak**	**+**	**0.50**	**Missed**	**− 0.23**	**0.1**
	2	29	+	Missed	Missed	0.34	14.43	0.08	0.19
	3	9	+	Weak	Missed	0.23	Missed	0.22	-0.02
	4*	**6**	**+**	**Missed**	**Missed**	**0.67**	**13.54**	**0.14**	**− 0.02**
	5	8	+	Weak	Missed	0.24	18.23	0.04	− 0.23
	6	11	+	Missed	Missed	0.40	18.70	0.01	− 0.01
TIM	1*	**58**	**+**	**Medium**	**+**	**0.55**	**46.85**	**− 0.08**	**− 0.61**
	2.1*	**10**	**+**	**Medium**	**+**	**0.67**	**21.06**	**0.42**	**− 0.02**
	2.2*	**2**	**+**	**Missed**	**Missed**	**0.47**	**14.93**	**0.36**	**− 0.24**
	3.1	6	+	Weak	Missed	0.56	20.59	1.45	− 0.23
	3.2	2	+	Missed	Missed	0.68	Missed	0.40	− 0.12
	4	26	+	Weak	Missed	0.51	11.23	0.54	− 0.70
	5	Missed	Missed	Missed	Missed	0.59	Missed	− 0.13	− 0.17
	6	Missed	Missed	Missed	Missed	0.64	Missed	− 0.14	− 0.15
PGM	1*	**32**	**+**	**Strong**	**+**	**0.37**	**39.78**	**1.01**	**− 0.41**
	2	9	+	Weak	Missed	0.67	16.89	0.08	0.12
	3	7	+	Weak	Missed	0.51	Missed	− 0.33	0.01

*Selected binding sites for docking calculations

As described in the Methods, following the final prioritization of allosteric sites, bacterial enzymes were compared with their human homologs to evaluate sequence and structural conservation within the putative allosteric pockets. Table 3 summarizes both local (binding-site) and global similarities, reported as percent sequence similarity and identity, along with RMSD values for the binding sites and overall structures. In aldolase, binding site #1 exhibited the greatest divergence from its human ortholog (PDB ID listed in Table 3), highlighting its strong potential for species-specific targeting. In contrast, binding site #4 showed moderate sequence similarity and identity, suggesting partial conservation that may limit selectivity and therefore warrants careful consideration during inhibitor design. For TIM, the evaluated sites exhibited moderate conservation, with sequence similarity of 60–70% and identity of approximately 50–60%. While not fully divergent, this level of conservation remains compatible with selective allosteric modulation, particularly when combined with differences in local pocket geometry and dynamics. Notably, PGM showed relatively low sequence and structural conservation at the selected allosteric site, supporting a high likelihood of species specificity. The pronounced divergence of this hinge-associated pocket, together with its strong dynamic and energetic coupling, makes it an especially promising target for selective antibacterial drug screening.

Despite the low RMSD values observed for the selected binding sites, the substantially higher global RMSD values between human and bacterial homologs (18–29 Å) indicate pronounced differences in domain organization, oligomerization state, and overall structural flexibility. Importantly, these global structural variations do not extend to the local architecture of the binding pockets, which remain highly conserved across homologs. This distinction suggests that the observed structural divergence arises primarily from differences in quaternary arrangement and conformational dynamics rather than from fundamental changes in the core fold or active site geometry.

**Table 3:** Sequence and structure-based comparison of bacterial and human homologs

	Aldolase PDB id: 4ALD		TIM PDB id: 1WYI		PGM PDB id: 1YFK
	B.Site # 1	B.Site # 4	B.Site # 1	B.Site # 2	B.Site #1
% Similarity	7.7	60.0	68.8	71.4	22.2
% Identity	3.8	60.0	62.5	57.4	11.1
RMSD	6.06	3.5	2.89	7.45	4.21
RMSD Total*	29.1		18.4		25.4

* Overall structural deviation obtained from global alignment of full-length protein structures

## PART II. Virtual screening of FDA drug compounds to putative allosteric sites

Structure-based molecular docking was conducted for each selected binding site across the ten glycolytic enzymes to identify FDA-approved drug candidates exhibiting consistently high predicted binding affinities. The binding sites of the remaining seven glycolytic enzymes had been previously characterized (see Fig. 1), and the amino acid residues defining all selected target sites are provided in Supplementary Table S3. Furthermore, the selected target sites were evaluated based on pocket descriptors, including volume, surface area, druggability, and DrugScore, as calculated using the CavityPlus tool, summarized in Supplementary Table S4 and illustrated in Supplementary Figure S1. Accordingly, nine out of the seventeen target sites were classified as druggable, five as moderately druggable, and three as undruggable. All cavities identified for aldolase were classified as undruggable. However, the allosteric assessment of each glycolytic enzyme, based on multiple criteria, indicated a strong allosteric capacity for aldolase. A similar observation was made for TIM, where the second binding site was classified as undruggable, yet exhibited strong allosteric features according to several evaluation tools.

**Fig. 6:**
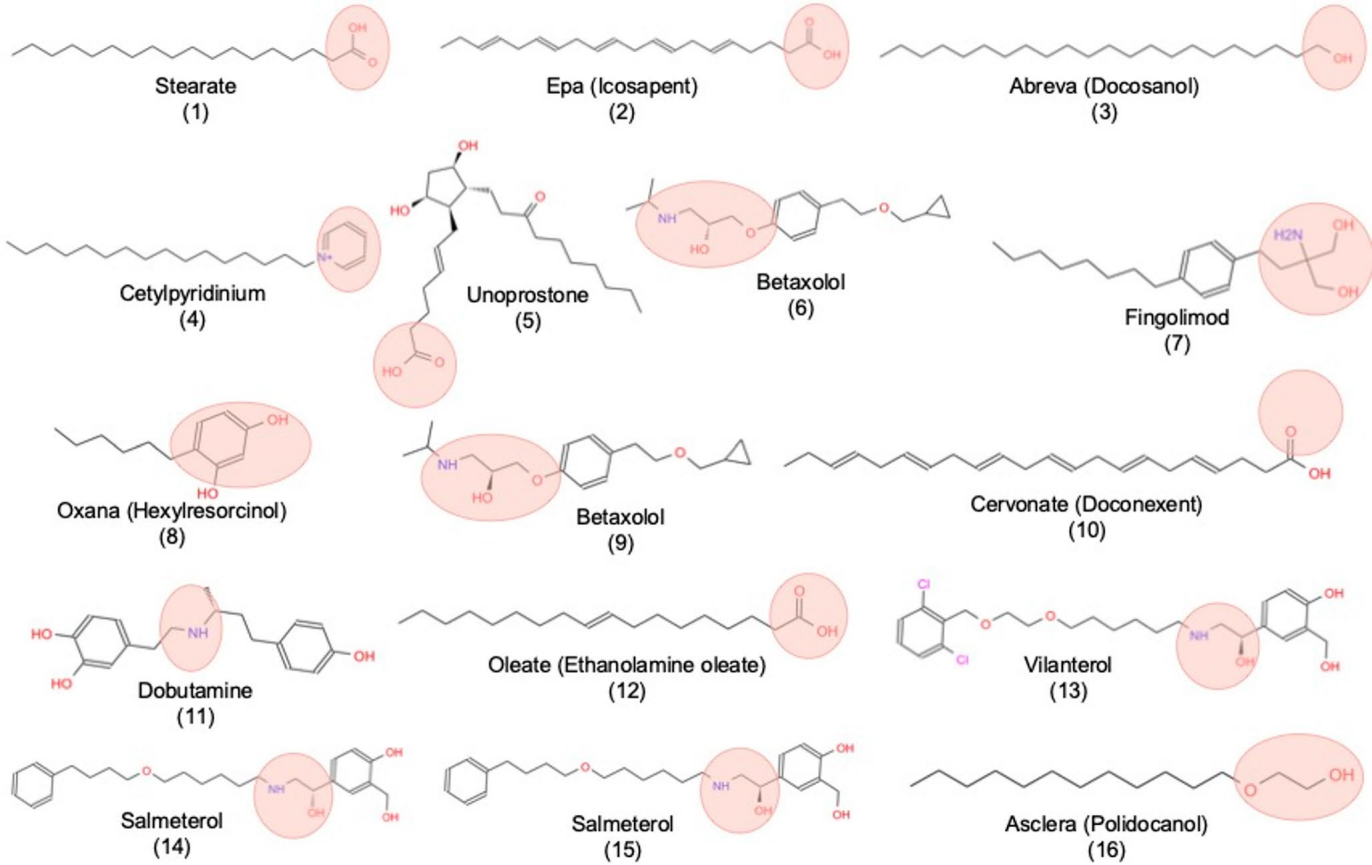
2D representation of sixteen compounds obtained using the top 20% of each screening result. Polar or ionizable groups are represented with pale pink circles

Docked poses were ranked using the ChemPLP scoring function. To reduce bias associated with molecular size and flexibility, docking scores ($\:S)$ were normalized with respect to ligand size, which is represented by the square root of the number of heavy atoms ($\:S/\sqrt{N}$). The square root normalization was chosen to correct for the well-known sublinear scaling of binding affinity with molecular size, arising from geometric saturation of protein–ligand interactions and steric constraints in the binding pocket; thus, a linear normalization by *N* would over-penalize larger ligands. This approach is consistent with ligand efficiency concepts, which normalize affinity by molecular size to account for diminishing energetic returns with increasing molecular weight [43] and provides a balanced correction that reduces size bias while preserving meaningful score differences. As shown in Supplementary Fig. 2, the correlation between docking score (S) and heavy atom count ($\:N$) decreased substantially after normalization ($\:{R}^{2}\:$from 0.68 to 0.02), indicating effective correction of size dependence. For each of the seventeen binding sites, the top 20% of ranked compounds (*n* = 323 per site) were retained for further analysis. Intersection analysis was subsequently performed to identify compounds common to all seventeen top-ranked subsets. The resulting consensus compounds are summarized in Table 4; Fig. 6.

**Table 4:** Top-ranked compounds with ZINC ID, drug name, chemical formula, molecular weight (MW), logP, number of hydrogen bond donor and acceptors (HBD/HBA) and number of heavy atoms

ID	ZINC ID	Drug name	Chemical formula	MW	Disease	logP	# of HBD	# of HBA	# Rot. Bonds
1	ZINC000004978673	Stearate	C18H36O2	284.5	**–**	6.33	0	2	16
2	ZINC000004474603	Epa (Icosapent)	C20H30O2	302.5	Autoimmune Disorder Rheumatoid Arthritis, Cancer Major Depressive Disorder Muscle Weakness	5.99	0	2	13
3	ZINC000006920384	Abreva (Docosanol)	C22H46O	326.6	Reccurent Herpes Labialis Oral Herpes Simplex	7.80	1	1	20
4	ZINC000006845963	Cetylpyridinium	C21H38N^+^	304.5	COVID-19 Gingivitis - Pregnancy	6.46	0	0	15
5	ZINC000008214703	Unoprostone	C22H38O5	382.5	Retinitis Pigmentosa Dry Age Related Macular Degeneration	4.26	2	5	15
6*	ZINC000001530568	Betaxolol	C18H29NO3	307.4	Glaucoma - Ocular Hypertension	2.39	2	3	11
7	ZINC000001542002	Fingolimod	C19H33NO2	307.5	Relapsing multiple sclerosis (rms)	3.20	3	2	12
8	ZINC000001576892	Oxana (Hexylresorcinol)	C12H18O2	194.3	Postoperative Sorethroat - Sore Throat	3.22	2	2	5
9*	ZINC000001530567	Betaxolol	C18H29NO3	307.4	Glaucoma - Ocular Hypertension	2.39	2	3	11
10	ZINC000004474564	Cervonate (Doconexent)	C22H32O2	328.5	Autoimmune Disorder, Rheumatoid Arthritis - Cancer Coronary Artery Disease	6.55	0	2	14
11	ZINC000000003911	Dobutamine	C18H23NO3	301.4	**–**	2.96	4	3	7
12	ZINC000006845860	Oleate (Ethanolamine oleate)	C18H34O2	282.5	Oesophageal varices haemorrhage	6.11	0	2	15
13	ZINC000003991624	Vilanterol	C24H33Cl2NO5	486.4	Asthma	4.61	4	5	16
14^**#**^	ZINC000003785268	Salmeterol	C25H37NO4	415.6	Chronic obstructive pulmonary disease - Asthma	4.11	4	4	16
15^**#**^	ZINC000003799072	Salmeterol	C25H37NO4	415.6	Chronic obstructive pulmonary disease - Asthma	4.11	4	4	16
16	ZINC000008437287	Asclera (Polidocanol)	C14H30O2	230.4	Varicose Veins of Lower Limb - Chronic Achilles Tendinopathy	3.92	1	2	13

* Two different isoforms of betaxolol

# Two different isoforms of salmeterol

**Fig. 7:**
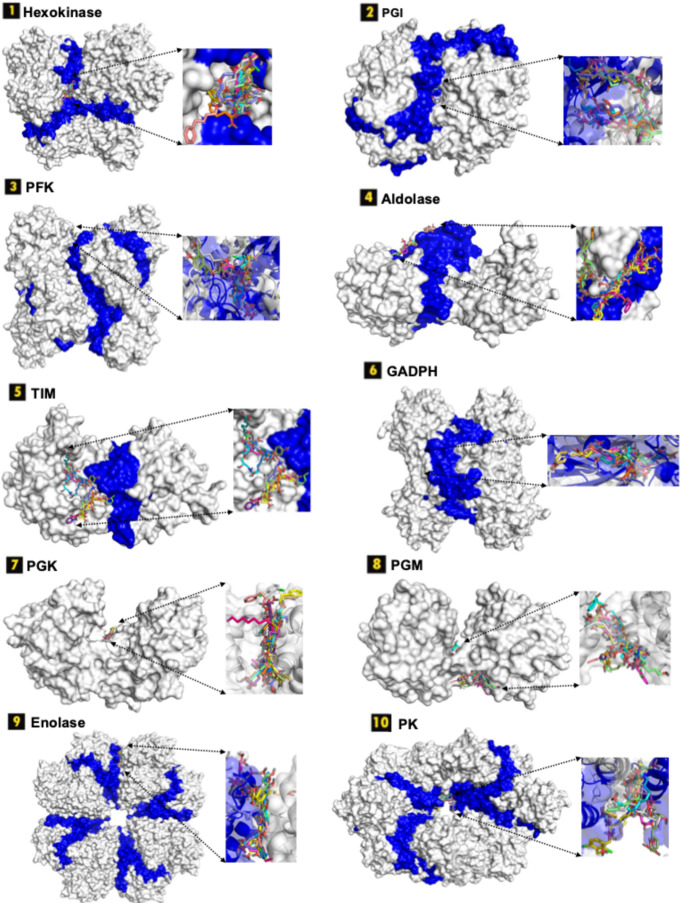
Docked poses of 16 common compounds on interface-centered allosteric sites across ten glycolytic enzymes. Interface regions are highlighted in dark blue

A striking outcome of this virtual screening study is the pronounced physicochemical convergence observed among top-ranking FDA-approved compounds across all ten glycolytic enzymes. Despite structural diversity among the enzymes and variation in the location of predicted allosteric sites, the highest-affinity ligands consistently exhibited amphiphilic architectures characterized by extended hydrophobic domains coupled to a single polar or ionizable anchoring group, as depicted in Fig. 6. Notably, these sites were predominantly localized at subunit interfaces or critical hinge regions—structural elements known to mediate conformational transitions and inter-domain communication (See Fig. 7). Unlike deeply buried orthosteric pockets, such regulatory regions typically present broad, partially solvent-exposed hydrophobic surfaces with limited geometric constraints, favoring ligands that maximize surface burial while retaining conformational adaptability. Given that several of the top-ranked compounds possess a high number of rotatable bonds, docking results were interpreted with consideration of ligand flexibility. Rather than relying solely on a single optimal pose, we focused on consistent pocket occupancy and binding site preference across targets, as flexible ligands may adopt multiple energetically similar conformations within a given binding region.

The recurrent enrichment of long-chain fatty acids, lipid-like molecules, and flexible β-agonist–like scaffolds (e.g., salmeterol, betaxolol, vilanterol, dobutamine, fingolimod) therefore suggests that allosteric modulation across glycolysis may be governed by a shared structural principle: stabilization or perturbation of hydrophobic interfacial packing rather than classical lock-and-key recognition. These findings indicate a unifying structural principle: glycolytic enzymes share conserved, interface-centered regulatory features that could be targeted to modulate metabolism across multiple enzymes.

As ligand binding affinity alone does not necessarily imply functional allosteric modulation, the AlloSigMA tool was employed to quantify the allosteric effects of each hit compound on the active site through residue-level free energy changes (ΔG). As summarized in Supplementary Figure S3, this analysis was restricted to pyruvate kinase (PK) and phosphofructokinase (PFK), as these were the only enzymes with experimentally characterized allosteric inhibitors. Accordingly, the sixteen compounds were evaluated in comparison with reference inhibitors bound at their experimentally validated allosteric sites (BS2 for PFK and BS1 for PK; see Fig. 1).

For PFK, all sixteen compounds, as well as the PK allosteric inhibitor IS130, exhibited mean ΔG values in the range of − 0.4 to − 0.2 kcal/mol (chain A), − 0.7 to − 0.3 kcal/mol (chain B), − 0.7 to − 0.3 kcal/mol (chain C), and − 0.1 to 0.3 kcal/mol (chain D). Except for chain D, all catalytic sites (chains A–C) showed consistently negative ΔG values, indicative of restricted dynamics upon allosteric ligand binding. Notably, these values were more negative than those observed for the native allosteric inhibitor PEP (− 0.2, 0.1, 0.1, and − 0.1 kcal/mol for chains A–D, respectively), suggesting a comparatively stronger allosteric coupling.

In contrast, for PK, IS130 displayed ΔG values close to zero (see Supplementary Figure S3b), whereas all sixteen compounds and PEP yielded moderately negative values ranging from − 0.3 to 0.0 kcal/mol (− 0.3 to − 0.1 for chain A, − 0.1 to 0.1 for chain B, − 0.1 to 0.0 for chain C and D). Overall, the allosteric effects observed for PFK were more pronounced than those for PK. Nevertheless, the consistently negative ΔG values across most compounds suggest that the identified hits can stabilize allosteric effects at the active site, supporting their potential as functional allosteric modulators, albeit with system-dependent magnitude.

**Fig. 8:**
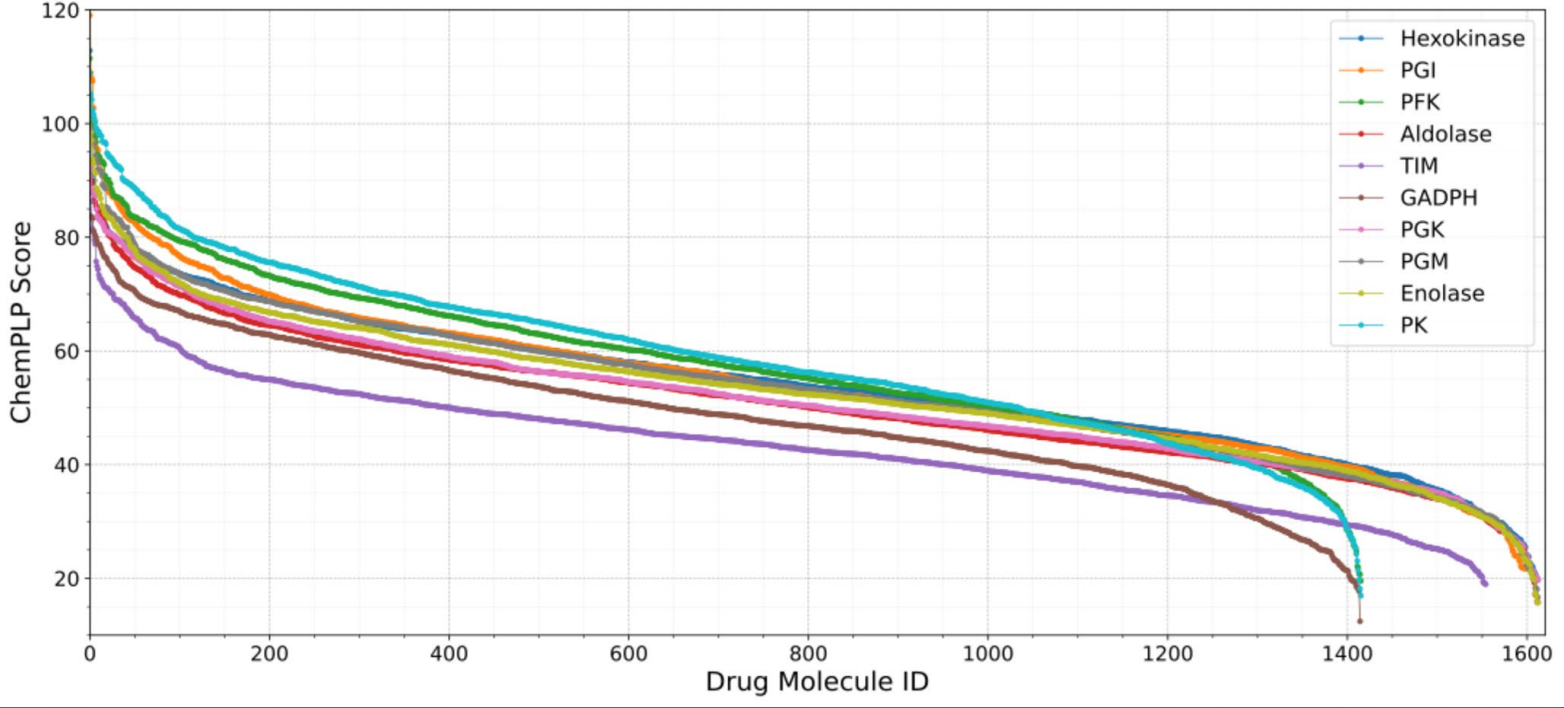
ChemPLP scores of 1,615 FDA-approved compounds docked into interface-located allosteric pockets across ten glycolytic enzymes

To further assess relative binding performance, ChemPLP scores of all 1,615 FDA-approved compounds were compiled across the 17 selected target sites and systematically compared (Fig. 8), enabling cross-target evaluation of predicted binding affinities. Strikingly, the highest score distribution was observed at the experimentally validated allosteric site of pyruvate kinase (top blue line in Fig. 8), reported to accommodate the allosteric inhibitor IS-130 [44]. The second-highest binding scores were associated with the experimentally characterized allosteric site of phosphofructokinase, which binds phosphoenolpyruvate (PEP) (green line in Fig. 8).

The predominance of experimentally confirmed allosteric pockets among the top-ranking sites is particularly noteworthy. Importantly, the highest binding score distributions were observed at experimentally validated allosteric sites in pyruvate kinase and phosphofructokinase (See Fig. 8), both of which are well-established model systems for allosteric regulation [6, 7, 39]. This agreement between computational predictions and well-characterized regulatory pockets provides indirect validation of both the binding site identification strategy and the docking protocol employed in this study. The ability to recover known allosteric hotspots supports the framework’s reliability when extended to less-characterized enzymes. These findings suggest that evolutionarily conserved regulatory cavities possess structural and physicochemical properties that inherently favor high-affinity ligand binding. In contrast, several computationally predicted pockets displayed comparatively attenuated score distributions. Collectively, these results not only validate the docking strategy but also underscore experimentally defined allosteric regions as structurally favored sites for drug repurposing and multi-target metabolic intervention.

Because interface docking can overemphasize hydrophobic contributions, residue types interacting with the ligand were identified for genuine hotspot recognition. As summarized in Tables 5 and 6, residues interacting with at least 12 out of 16 drug candidates were classified into hydrophobic and polar categories. Across the 10 allosteric sites of different enzymes included in the docking analysis, a total of 35 polar residues were identified at the interaction interfaces, compared to 30 hydrophobic residues. Notably, certain polar residues—particularly *Arg*, *Glu*, *Ser*, and *Thr*—were observed more frequently than others, suggesting a substantial contribution of hydrogen bonding and electrostatic interactions to ligand stabilization. The recurrent presence of charged residues such as *Arg* and *Glu* indicates that salt bridges and long-range electrostatic interactions may play an important role in binding.

Although *Ile* was the most frequently observed hydrophobic residue, the overall distribution of interacting residues does not indicate a clear dominance of hydrophobic contacts. Instead, the comparable—and slightly higher—representation of polar residues suggests that binding within these allosteric pockets is not primarily driven by hydrophobic interactions. This observation argues against a strong hydrophobic bias in the ChemPLP scoring function under the present docking conditions. It supports a balanced contribution of polar and nonpolar interactions in ligand recognition. Moreover, no significant correlation was observed between ligand hydrophobicity (logP) and docking score, further supporting the absence of systematic hydrophobic overestimation.

**Table 5:** Residue types and IDs interacting with at least 12 out of 16 FDA compounds

Site ID	Residue Type/ID	
	Polar	Hydrophobic
1	Asp299/Glu49/Thr46	Pro301/Trp311/Leu300/Leu302/Phe50
2	Arg89/Glu93/Glu107/Ser98	Tyr119/Val109/Phe99/Phe110/Ile92/Ile108
3	Arg632/Lys574/Lys567/Gln568/Ser569/Ser571	Phe578
4	Arg245/Glu253	Tyr255/Ala241
5	Asn21	Leu248
6	Arg53/Glu204/Ser281/Thr49	Gly52/Met50/Val284
7	Arg36/Gln39/Asn35/Glu365/Ser372/Thr373/Thr369	Ile360/Ile371
8	Arg89	Tyr84/Leu315/Val313/Val83
9	Lys362/Glu358	Tyr430/Ala363/Met359/Ile332
10	Asn369/His365/Ser362/Thr366/Thr353	Ala358/Ile361

**Table 6:** Number of times a residue type is observed in an allosteric site interacting with at least 12 out of 16 FDA compounds

Site ID	Polar									Hydrophobic										
	R* Arg	K Lys	D Asp	Q Gln	*N* Asn	E* Glu	H His	S* Ser	T* Thr	*P* Pro	Y Tyr	C Cys	G Gly	A Ala	M Met	W Trp	L Leu	V Val	F Phe	I* Ile
1			1			**1**			**1**	1						1	2		1	
2	**1**					**2**		**1**			1							1	2	**2**
3	**1**	2		1				**2**											1	
4	**1**					**1**					1			1						
5					1												1			
6	**1**					**1**		**1**	**1**				1		1			1		
7	**1**			1	1	**1**		**1**	**2**											**2**
8	**1**										1						1	2		
9		1				**1**					1			1	1					**1**
10					1		1	**1**	**2**					1						**1**
Total	**6**	3	1	2	3	**7**	1	**6**	**6**	1	4	0	1	3	2	1	4	4	4	**6**

* Residues in bold type interact with at least 6 out of 10 binding sites located at the interface

The amphiphilic nature and extensive hydrophobic domains of the 16 candidate compounds are likely to enhance surface burial, facilitating their adaptation to the large, solvent-exposed architecture of allosteric binding pockets. In addition, our observation that polar residues such as *Arg*, *Glu*, *Ser*, and *Thr* play a critical role in enzyme–ligand interactions suggests that allosteric modulation is mediated by a balance between hydrophobic stabilization and specific polar contacts. Furthermore, the number of hydrogen-bond donors ranged from 0 to 4, while hydrogen-bond acceptors ranged from 0 to 5. These findings are consistent with previous reports, including the review by Tee and Berezovsky [45, 46], which indicate that allosteric ligands tend to possess relatively fewer hydrogen-bond donors and acceptors.

**Fig. 9:**
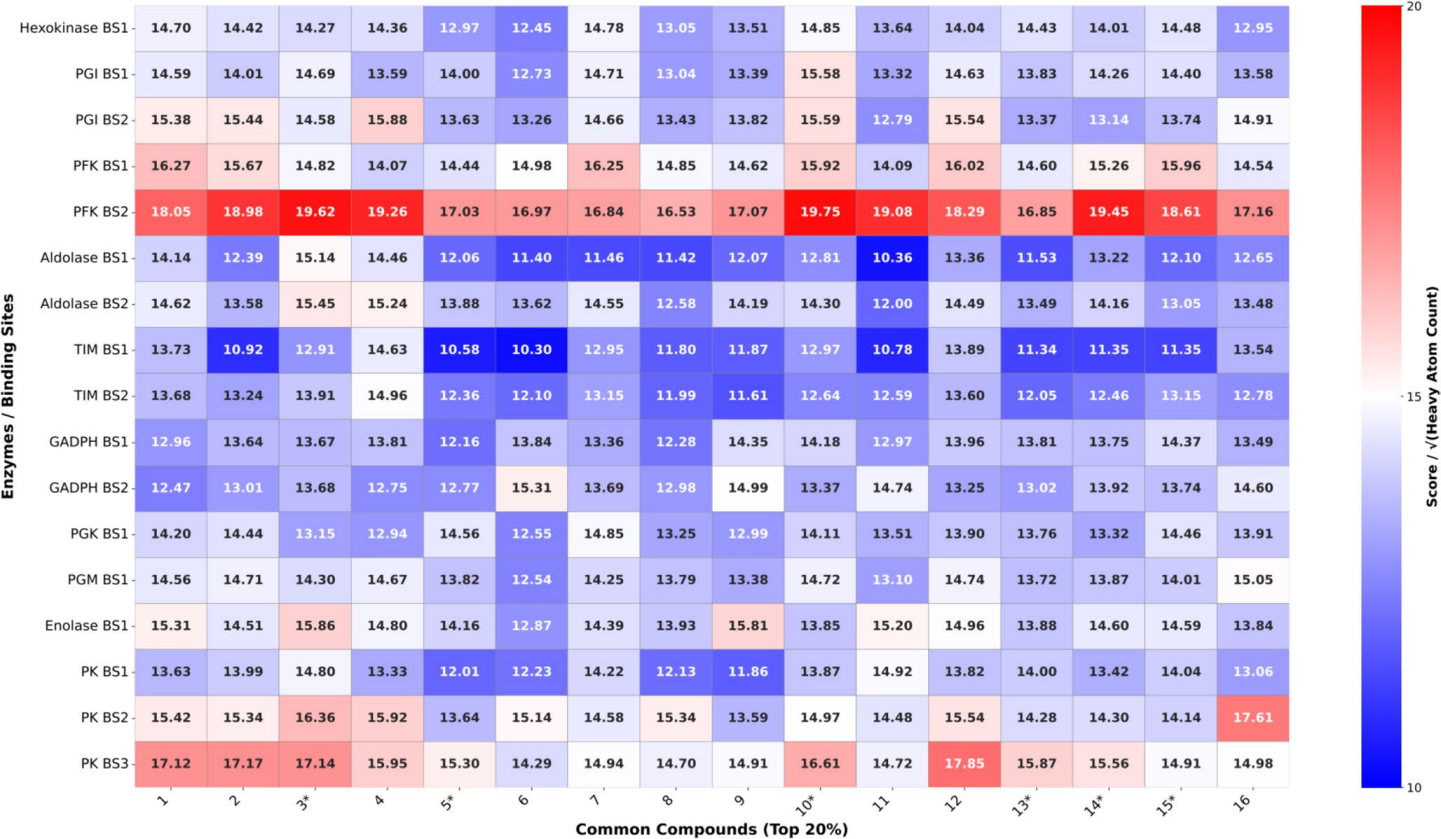
Heat map representation of score values of 16 FDA compounds common in 17 identified potential allosteric sites. Stars indicate common compounds in two sets with normalized and unnormalized score values. See text for details

The heat map of ChemPLP scores provided in Fig. 9 highlights differences in predicted binding affinities across seventeen target sites from ten glycolytic enzymes. PFK BS2 exhibits the highest scores across multiple compounds (18–19.75), corresponding to its experimentally validated allosteric site, while PK BS3 also shows consistently high scores (15.95–17.85), indicating a favorable regulatory site. Several other sites, including TIM BS1/BS2 and Aldolase BS1, display lower scores (10–13), suggesting limited binding potential. Overall, the heat map identifies the most promising allosteric and regulatory sites, providing a focused framework for targeted compound screening and drug design efforts.

The same filtering protocol was rigorously applied to the unnormalized docking scores of all 1,615 compounds to ensure methodological consistency. As demonstrated by the heat map (Supplementary Figure S4), 45 compounds reproducibly ranked within the top 20% across all seventeen target sites, underscoring the robustness of the selection criterion.

Importantly, cross-comparison of the two hit compound sets identified six overlapping candidates, which were consistently selected under both scoring schemes and are explicitly highlighted (starred) in Fig. 9 and Supplementary Figure S4. These correspond to compounds 3, 5, 10, 13, 14, and 15 in Table 4, namely Abreva, Unoprostone, Cervanote, Vilanterol, and Salmeterol (represented by two isoforms). The convergence of these compounds across independent scoring approaches further supports their prioritization as robust hits.

**Fig. 10:**
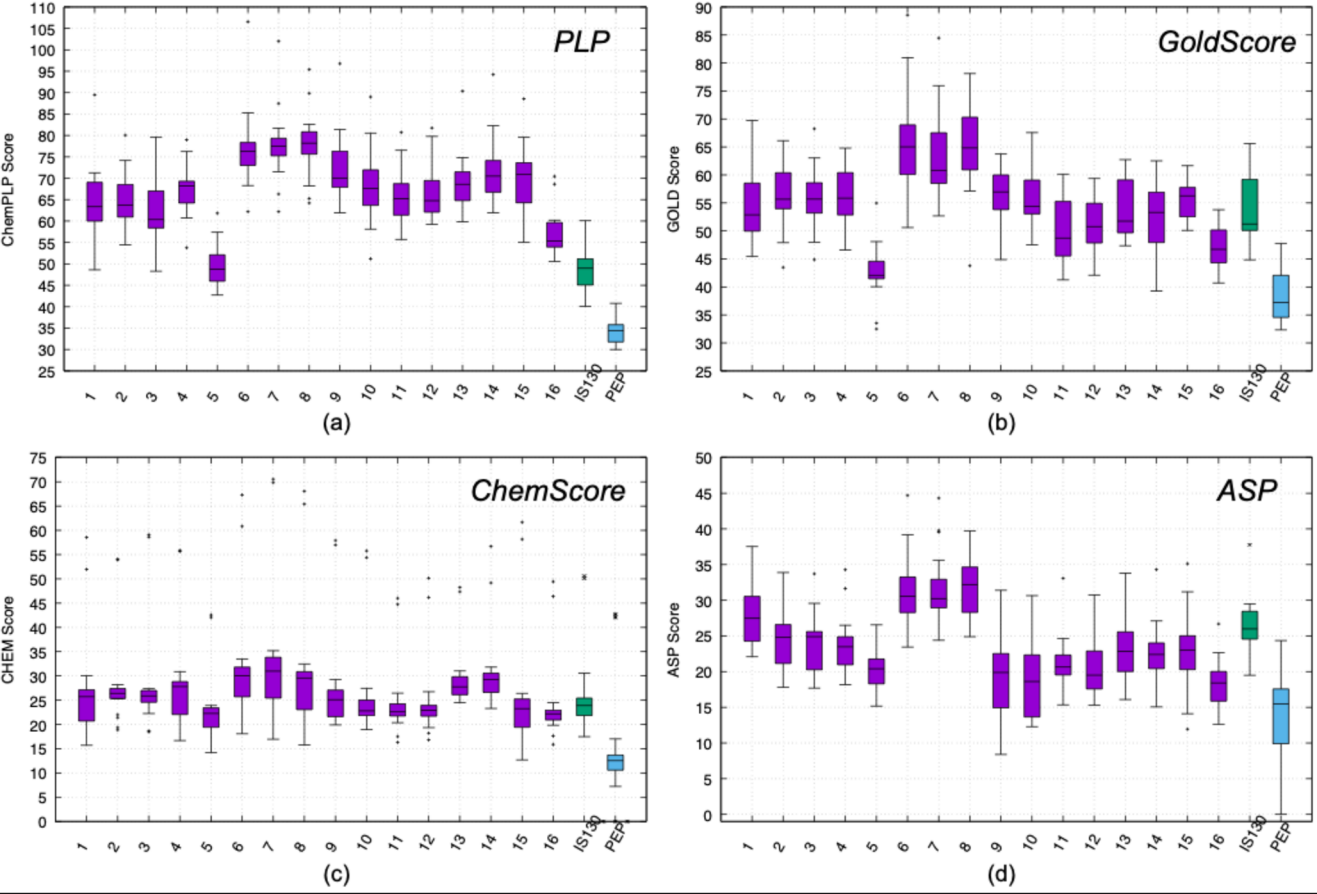
Distributions of score values of 16 hit compounds, and two experimentally reported allosteric inhibitors IS130 and PEP docked using GOLD software tool [40, 41] via **a** ChemPLP, **b** GoldScore, **c** ChemScore and **d** ASP scoring functions

To evaluate the robustness of our findings, the final set of sixteen compounds was re-docked to all seventeen target sites using the GoldScore, ChemScore, and ASP scoring functions. In addition, the experimentally validated allosteric inhibitors IS130 (pyruvate kinase) and PEP (phosphofructokinase) were also re-docked to enable a comparative assessment. The resulting score distributions for the sixteen compounds, IS130, and PEP across all seventeen allosteric sites were presented as a bar plot in Fig. 10. Accordingly, all sixteen hit compounds consistently achieved higher scores than the allosteric inhibitor PEP across all scoring functions. In contrast, IS130 exhibited comparable performance to the hit compounds in GoldScore, ChemScore, and ASP; however, it showed noticeably lower values under the ChemPLP scoring function, suggesting a less favorable ranking by this scoring scheme.

A common feature of our sixteen hit compounds is the presence of flexible long aliphatic tails, which can increase conformational freedom and therefore make accurate pose prediction and ranking more challenging. Despite this, the ChemPLP scoring function has been widely reported to show strong performance in both pose reproduction and ligand ranking across chemically diverse and flexible datasets, largely due to its piecewise linear potential and efficient optimization strategy that balances computational efficiency with effective treatment of steric and hydrogen-bonding interactions.

In this context, the consistent performance trends observed in our dataset further support the robustness of the docking protocol applied in this study. Nevertheless, we acknowledge that even well-validated scoring functions, including ChemPLP, have intrinsic limitations in fully capturing the entropic and dynamic effects associated with highly flexible ligands, and some level of uncertainty in absolute ranking remains unavoidable.

Finally, to directly assess the species-specificity of the identified allosteric sites, all sixteen compounds, together with IS130 and PEP, were re-docked to the corresponding homologous sites in the human proteins. Importantly, human hexokinase could not be included in this analysis because the available structure (PDB ID: 4DHY) represents a monomeric form lacking a structurally equivalent allosteric pocket; thus, no meaningful cross-species comparison is possible for this enzyme. From the resulting plot (See Suppl Figure S5), all hit compounds consistently exhibited lower score values at the human homologous sites compared with the corresponding target sites identified in S.*aureus* species. Therefore, these findings provide compelling and internally consistent evidence that the identified binding sites—and the associated hit compounds—are intrinsically species-specific. This effectively supports the conclusion that these compounds preferentially target bacterial enzymes with minimal likelihood of interaction with the human counterparts.

## Conclusions

This study demonstrates the feasibility of selectively targeting bacterial glycolysis through pathway-wide allosteric modulation. Despite the increasing interest in allosteric drug discovery, experimentally validated allosteric binding pockets remain available only for a limited subset of glycolytic enzymes, most notably phosphofructokinase and pyruvate kinase, where ligand binding and regulatory effects have been extensively characterized using structural, kinetic, and biophysical approaches [6, 7, 44]. In contrast, for the majority of enzymes within the glycolytic pathway, including aldolase, triosephosphate isomerase (TIM), phosphoglycerate mutase (PGM), phosphoglycerate kinase (PGK), and enolase, regulatory behavior is primarily inferred from conformational dynamics, inter-subunit communication, or oligomerization effects rather than direct experimental identification of ligand-binding allosteric sites [8–10]. This lack of comprehensive experimental characterization highlights a critical gap in our understanding of pathway-wide allosteric regulation.

In this context, the present study is designed as a systematic, hypothesis-generating framework to identify and prioritize latent allosteric regions across the entire glycolytic pathway [47]. By integrating complementary computational approaches capturing structural, dynamic, network-based, and energetic features, our methodology enables the identification of biologically plausible regulatory sites even in the absence of experimentally resolved allosteric pockets. The convergence of multiple independent methods, together with the consistent localization of predicted sites at interfacial and hinge regions known to mediate protein regulation, supports the robustness of the identified targets.

By integrating elastic network models, residue interaction networks, and machine-learning approaches, we systematically identified high-confidence allosteric sites across all ten *Staphylococcus aureus* glycolytic enzymes. Our analyses revealed diverse structural features, from interfacial hub regions in oligomeric enzymes to hinge-driven, dynamics-dependent allosteric effects in monomeric enzymes, highlighting the structural and mechanistic heterogeneity underlying allosteric regulation. Comparative evaluation with human homologs based on sequence and structure confirmed pronounced species-specific divergence in key pockets, supporting selective inhibition with minimal off-target effects.

Virtual screening of 1,615 FDA-approved compounds identified sixteen multi-target candidates with strong binding affinities to conserved regulatory sites across the pathway. These compounds consistently exhibited amphiphilic, interface-stabilizing architectures, suggesting that glycolytic enzymes harbor evolutionarily conserved amphiphile-responsive surface cavities that can be used for pharmacological intervention. The convergence of predicted binding preferences across all ten enzymes highlighted the dominant abundance of polar residues *Arg*, *Glu*, *Ser*, and *Thr* alongside the hydrophobic residue *Ile*, which interact with at least 75% of the candidate compounds. This finding underscores the potential of network-informed polypharmacology to simultaneously disrupt multiple glycolytic nodes, offering a robust strategy for metabolic intervention.

While the present study provides a comprehensive computational framework for identifying and prioritizing allosteric sites, experimental validation remains an essential next step. Future work will focus on targeted validation of selected high-confidence sites, including mutagenesis experiments to probe functional relevance and biophysical assays such as surface plasmon resonance (SPR) or isothermal titration calorimetry (ITC) to confirm ligand binding. Especially, interface- and hinge-associated sites identified in enzymes such as triosephosphate isomerase and phosphoglycerate mutase represent promising candidates for experimental investigation. These efforts will further elucidate the relationship between ligand binding and functional allosteric modulation, ultimately enabling structure-guided optimization of multi-target inhibitors. Moreover, by framing glycolysis as an interconnected regulatory network rather than a linear sequence of isolated enzymes, this study establishes a blueprint for rational, species-specific antimicrobial design, providing a promising avenue to overcome compensatory metabolic adaptations and mitigate antibiotic resistance.

## Supplementary Information

Below is the link to the electronic supplementary material.

Supplementary Material 1

## Data Availability

The complete docking score dataset for all 1,615 FDA-approved compounds across the seventeen binding sites has been provided as an Excel file and made available in a GitHub repository (https://github.com/sudevurall/10Enzyme17BindingSites).
