# Supplementary material for: Breaking glycolysis: allosteric hotspots for multi-target drug repurposing
Source: J Comput Aided Mol Des. 2026 May 16;40(1):124. doi: 10.1007/s10822-026-00830-5 (PMC13179880; doi:10.1007/s10822-026-00830-5)
Supplement: Supplementary file 1 — Supplementary Material 1 [file 10822_2026_830_MOESM1_ESM.docx]

**SUPPORTING INFORMATION**

## Breaking Glycolysis: Allosteric Hotspots for Multi-Target Drug Repurposing

## Latife Sude Vural^1^, Elcin Kahraman^2^, Simay Mintemur^2^, Sinem Urhan^2^, E. Demet Akten^2*^

*^1^Graduate Program of Computational Sciences and Engineering, Graduate School of Science and Engineering, Kadir Has University, Istanbul, Turkey*

*^2^Department of Molecular Biology and Genetics, Faculty of Engineering and Natural Sciences, Kadir Has University, Istanbul, Turkey*

^*^Corresponding Author

[demet.akten@khas.edu.tr](mailto:demet.akten@khas.edu.tr)

+90 212 533 65 32 (x1350)

<https://orcid.org/0000-0002-0358-3171>

**Supplementary Table 1**. Binding site residues for all identified binding sites, which are evaluated for allosteric likelihood in Fructose 1,6-Bisphosphate Aldolase, TIM, and phosphoglycerate mutase.

| **Site ID** | **Aldolase** | **TIM** | **PGM** |
| --- | --- | --- | --- |
| 1 | **A:**L3-S5/I97/G100-T102/  K130-V132 | **A:**A49-I52/L54/A73-Y77/F84-T88/L93/R108  **B:**A49-A51/A73-Y77/F84-T88/R108 | V75/R81-I86/G89/I311-Q316/506 |
| 2 | **A:**P1/E8-M9/D12/Y18/P44-I46/I78-A81/R284-H287 | **BS2.1.B:**K170-V173/A215-R217  **BS2.2.B:**S8/R10-P12/M233-T236/I238-D239 | G22-A24/P31/F33-D34/  N38/P41/I45/V470/L472 |
| 3 | **A:**S109/S111-F113/N116/  L138-T140/I154/E160 | **BS3.1.A:**S8/R10/P12/M233-T236/I238-D239  **BS3.2.A:**Y100/L169-V173/A215-R217 | N172/I176/F179/Y186-A188/A222-E226/N246/  D249 |
| 4 | **A**:N27/F30/Q237/S240-A241/V244/L261  **B:**Y255-R258/Y260-L261 | **A:**G104-S106/R109-E110/C136-T140/E143/V152/Q156/E177/I179 |  |
| 5 | **B:**M9/D12/Y18/P44/P79/  R284-K286 | **A:**L57-T58/V61-K62/G68-I71/L96-K99 |  |
| 6 | **B:**I97/G100-T102/K130-V132 | **B:**T58//V61-K62/G68-I71/L96-K99 |  |

**Supplementary Table S2.** Substrate and cofactor binding site residues for all ten enzymes

| **Druggable Site ID** | **Substrate** | **Cofactor** |
| --- | --- | --- |
| 1. Hexokinase | **A.B.C.D:**R109/D241/I265/G267-G268/  D434-S436/D470-G473 | **A.B.C.D:**D17-I19/L40/I42/K397/E400-S401/  N404-R405/W443/K446-R447/E450 |
| 2. PGI | **A.B:**K139**/**E145/R202/E285/K289/H306/S307/Y419/K420 |  |
| 3. PFK | **A:**G11/R72/I128/D129/M171-R173/E224/  H251/R254;**D:**R164,R245  **B**:R72/T127-D129/D131/M171-R173/E224/  H251/R254;**C:**R245  **C**:R72/T127-D129/M171-R173/E224/H251/  R254;**B:**R245  **D:**G11/D12/R72/I128/D129/M171-R173/  H251/R254;**A:**R164/R245 | **A**:G11/R72-P74/K77/G102-S105/R107/T127/D129/D131/R173  **B**:G11/C73/P74/K77/G102-S105/R107/D129/D131/R173  **C**:G11/R72-P74/K77/G102-S105/R107/D129/D131/R173  **D**:G11/R72-P74/K77/G102-S105/R107/D129/D131/R173 |
| 4. Aldolase | **A.B:**D85-H86/E137/D144/H181/H209-G210/N233 |  |
| 5. TIM | **A:**K19/H105/E177/I182/G221-K225/L242-A246  **B:**K19/I182/G221-K225/L242-A246 |  |
| 6. GADPH | **A:**S150-T152/H178/T181/T211/R234/Y314/  N316  **B:**S150-T152/H178/T181/T211/R234/Y314  **C:**S150-T152/T176/H178/T181/T211/R234/  Y320  **D:**S150-T153/T176/H178/T181/T211/N316/  Y320 | **A:**N8-I13/N33-L35/E77/P78/C96-T101/  S120/A121/S151/T181/N316/E317/Y320;**D:**P190  **B:**N8-I13/N33-L35/E77/P78/C96-T101/  S120/A121/S151/T181/N316/E317/Y320;**C:**P190  **C:**N8-I13/N33-L35/E77/P78/C96-T101/  S120/A121/S151/T181/N316/E317/Y320;**B:**P190  **D:**N8-I13/N33-L35/E77/P78/C96-T101/  S120/A121/S151/T181/N316/E317/Y320;**A**:P190 |
| 7. PGK | D21/N23/H59/K62/R119/G152-T153/H155-R156/H158/G374-A377 | G199-A201/D205-K206/G224/G225/Y228/G294-M295/N318-E325/G351-A356 |
| 8. PGM | E56/N61-E63/G65-I69/A71-G72/E87-D88/  K127-A131/L133/K162/S264-E265/  V463-I464 |  |
| 9. Enolase | **A.C.E.G:**E166/E207/D244/E291/D318/L341/K343/S370-S373/K394  **B.D.F.H:**S39/E166/E207/D244/E291/D318/L341/K343/H371-G374/K394 |  |
| 10. PK | **A.B:**K219/E221/M240-M246/A276/T277  **C.D:**R32/K219/V241-M246/A276/T277 | **A**:T9/I10/R32/N34/S36-G38/E42/H43/R46/D245/  S311/G312/A315  **B:**T9/I10/R32/N34/S36-G38/E42/H43/R46/S311/  G312/A315  **C:**R32/N34/S36-G38/E42/H43/R46/D245/S311  **D:**R32/N34-G38/E42/H43/R46/D245/S311 |

**Supplementary Table S3.** List of all residues observed in each selected druggable site for docking across all 10 glycolytic enzymes [11,14].

| **Druggable Site ID** | ***BS #1*** | ***BS #2*** | ***BS #3*** |
| --- | --- | --- | --- |
| 1.Hexokinase | **A:**L307/D310-R318  **B:**V37-V51/N53/Y56/E57/S78/  F296-P304/D306/L307/D310/S317/  L320/R405/L409  **C:**Y56-E57/K59-G61/E63-P69/  L71/I73-C77/K281-K283/K298 |  |  |
| 2. PGI | **A:**Y85/L86/R89/I92/T96/S97/F99/E107-F110/N113/H114/Y119/P270/N300/  T303/D304/  **B:**Y85/L86/R89/I92/T96/S97/F99/Y105-F110/N113/H114/A217/P270/Y274/  N300/T303/D304 | **B:**W47/L50-V63/G87/A91/I92  /Y173/F193-D197/V205-L211  /I214/A215/I219-A223/I226-K230/E385/E386/G389/Y390/  V392/Y393/E396 |  |
| 3. PFK | **A**:N130/D136-T143/L145/N146/W181/  T259-D262/V264-A266/R268/I288-N291  **B:**F137-T143/L145/N146/W181/T259-D262/V264-A266/R268/I288/N291  **C:**N130/F137-T143/L145/N146/W181/  T259/G260-D262/V264/A266/R268/  I288/N291  **D:**N130/F137-T143/W181/T259-D262/V264-A266/R268/I288/N290/N291 | **A:**R21/R25/D59/R156/V186-E189/K215/I322  **B:**R21/R25/D59/R156/V186-E189/K215/I322  **C:**R21/R25/D59/R156/V186-E189/K215/I322  **D:**R21/R25/D59/R156/V186-E189/K215/I322 |  |
| 4. Aldolase | **A:**L3-S5/I97/G100-T102/K130-V132 | **A**:N27/F30/Q237/S240-A241/V244/  L261  **B:**Y255-R258/Y260-L261 |  |
| 5. TIM | **A:**A49-I52/L54/A73-Y77/F84-T88/L93/  R108  **B:**A49-A51/A73-Y77/F84-T88/R108 | **BS2.1.B:**K170-V173/A215-R217  **BS2.2.B:**S8/R10-P12/M233-T236/I238-D239 |  |
| 6. GADPH | **A**:D48-R53/Y180/A203-N205/P236-A238  **B:**D48-M50/A203-N205/P236/T239/S281/D282/V284  **C:**A203/E204/P236  **D:**D48-G52/A203/E204/P236/S281-V284 | **A**:D48-R53/Y180/A203-N205/P236-A238  **B:**D48-M50/A203-N205/P236/T239/S281/D282/V284  **C:**A203/E204/P236  **D:**D48-G52/A203/E204/P236/S281-V284 |  |
| 7. PGK | N35/Q39/L175/K178-E179/ I360/E365-T373 |  |  |
| 8. PGM | V75/R81-I86/G89/I311-Q316/506 |  |  |
| 9. Enolase | **B:**V90/L94/L303/T306-D311/N324L330/  I332-N338/I347/T351/E352/D355/A356/  E358-A360/L362-Y365/G391/D424/I426-L434  **E:**T306-D311/T325/E326/A329/L330/I332-N338/D355/A356/E358-A360/L362-Y365/  G391/Y420-D424/I426-L434 |  |  |
| 10. PK | **A:**K260/R264/N267/N299/Y302-G304/  A337-Y340/K342/L343/D346/R347  **B:**K260/N299/Y302/D303/A337-Y340/  K342/L343/D346  **C:**K260/R264/N267/N299/Y302/D303/  D346/R347  **D:**Y302/Q338-Y340/K342/L343 | **A**:T353/A358/I361/S362/H365/T366/N369  **B:**T353/A358/I361/S362/H365/T366/N369/L370  **C:**T353/A358/I361/S362/H365/T366/N369  **D:**A358/I361/S362/H365/T366/N369/L370 | **A:**K271  **B:**G481/G483/  R484/A572-Q574 |

**Supplementary Table S4.** List of pocket descriptors for all seventeen target sites used for screening experiments.

| **Binding Site** | **Surface Area (Å²)** | **Volume (Å³)** | **DrugScore** | **Druggability** |
| --- | --- | --- | --- | --- |
| Hexokinase BS1 | 9151 | 20590.75 | 7636 | Druggable |
| PGI BS1 | 2580.25 | 3709.5 | 790 | Druggable |
| PGI BS2 | 123.75 | 75.38 | 252 | Less druggable |
| PFK BS1 | 5411.25 | 9427.25 | 3368 | Druggable |
| PFK BS2 | 5411.25 | 9427.25 | 3368 | Druggable |
| Aldolase BS1 | 281 | 264.5 | -852 | Undruggable |
| Aldolase BS2 | 153 | 172.62 | -997 | Undruggable |
| TIM BS1 | 617.25 | 455.88 | -101 | Less druggable |
| TIM BS2 | 539 | 703.25 | -712 | Undruggable |
| GADPH BS1 | 2554.25 | 4065.12 | 645 | Druggable |
| GADPH BS2 | 2554.25 | 4065.12 | 645 | Druggable |
| PGK BS1 | 1089.75 | 2357.5 | 65 | Less druggable |
| PGM BS1 | 1815 | 4101.25 | 2501 | Druggable |
| Enolase BS1 | 1571 | 2989.38 | 690 | Druggable |
| PK BS1 | 1278.75 | 2101.5 | 647 | Druggable |
| PK BS2 | 1083.5 | 2368.12 | 516 | Less druggable |
| PK BS3 | 1660.25 | 3054 | 129 | Less druggable |

**Supplementary Figure S1.** Binding pockets detected by CavityPlus.

**Supplementary Figure S2.** Correlation between ChemPLP scores and the square root of the total number of heavy atoms. Blue: raw ChemPLP scores plotted against the square root of heavy atom counts. Yellow: ChemPLP scores normalized by the square root of the total number of heavy atoms, plotted against the same.

(a)

(b)

**Supplementary Figure S3.** AlloSIGMA Results of sixteen hit compounds, IS130 and PEP for (a) phosphofructokinase (PFK) and (b) pyruvate kinase (PK) enzymes. ΔG values are averaged over all binding site residues in the catalytic site for each chain.

**Supplementary Figure S4.** Heat map representation of score values of 16 FDA compounds common in 17 identified potential allosteric sites. Stars indicate common compounds in two sets with normalized and unnormalized score values. See text for details.

**Supplementary Figure S5.** Distributions of ChemPLP score values of 16 hit compounds, and two experimentally reported allosteric inhibitors IS130 and PEP docked to corresponding sites in human homologous enzymes.
